# *Streptococcus thermophiles* DMST-H2 Promotes Recovery in Mice with Antibiotic-Associated Diarrhea

**DOI:** 10.3390/microorganisms8111650

**Published:** 2020-10-24

**Authors:** Jin-Shuang Hu, Yan-Yan Huang, Jia-Hua Kuang, Jia-Jia Yu, Qin-Yu Zhou, Dong-Mei Liu

**Affiliations:** School of Food Science and Engineering, South China University of Technology, Guangzhou 510640, China; 17862700663@163.com (J.-S.H.); 201810106521@mail.scut.edu.cn (Y.-Y.H.); kuangjiahua123@163.com (J.-H.K.); 201921025937@mail.scut.edu.cn (J.-J.Y.); 201920124874@mail.scut.edu.cn (Q.-Y.Z.)

**Keywords:** *Streptococcus thermophiles*, antibiotic-associated diarrhea, intestinal microbiota

## Abstract

Antibiotic-associated diarrhea (AAD) is the most common side effect of antibiotics and is routinely treated with probiotics in clinical. *Streptococcus thermophiles*, extensively utilized for producing dairy foods, has recently been regarded as a new promising probiotic candidate. In this study, the efficacy of *Streptococcus thermophiles* DMST-H2 (DMST-H2) for AAD treatment in mice was investigated. DMST-H2 was isolated from Chinese traditional yogurt, proved to be non-toxic, and presented tolerance against simulated gastrointestinal conditions *in vitro*. Additionally, genomic analysis revealed that it possessed genes related to acid tolerance, bile salt tolerance, adhesion, oxidative stress and bacteriocin production. The animal experiment results showed that both DMST-H2 treatment and natural recovery could reduce fecal water content. Compared with spontaneous recovery, DMST-H2 accelerated the recovery of the enlarged caecum and intestinal barrier injury from AAD, and further decreased endotoxin (ET), D-lactate (D-LA) and diamine oxidase (DAO) content in serum. Moreover, pro-inflammatory cytokines (TNF-α) were reduced, while interferon-γ (IFN-γ) and anti-inflammatory cytokines (IL-10) increased after treating with DMST-H2. Furthermore, DMST-H2 better restored the structure of intestinal flora. At the phylum level, Firmicutes increased and Proteobacteria decreased. These findings indicate that DMST-H2 could promote recovery in mice with antibiotic-associated diarrhea.

## 1. Introduction

Antibiotic treatment often causes diarrhea, which is called antibiotic-associated diarrhea (AAD). Various antibiotics result in AAD, especially aminopenicillins, cephalosporins, and clindamycin [1]. According to statistics, approximately 5% to 39% of patients who received antibiotic treatment might have mild to moderate diarrhea [2,3]. The major mechanisms of AAD include damage to the intestinal barrier, effects on immune homeostasis [4], disruptions to the normal composition of the gut microbiome [5], and alterations to intestinal metabolites [6].

Probiotics are non-pathogenic living microorganisms intended to colonize the intestinal tract and confer benefits on the host if given adequate amounts [7]. Probiotics have to resist low pH and bile salts to survive through the gastrointestinal tract. The purpose of probiotics is to exert beneficial health effects in vivo [8]. Probiotic intervention is now becoming a hot topic of research due to the encouraging effects of gastrointestinal diseases, such as ulcerative colitis, travelers’ diarrhea, and irritable bowel syndrome [3,9]. Meta-analyses about the effects of probiotics on AAD have emerged in large numbers. A meta-analysis including 17 randomized controlled trials with 3631 participants found that 8.0% of the probiotic group presented with AAD while the control group had 17.7%, and the probiotic strains *Lactobacillus rhamnosus* GG and *S. boulardii* showed similar results [3]. A network meta-analysis including ten probiotic intervention strategies revealed that *Lactobacillus rhamnosus* GG had the best prevention effectiveness and tolerance on AAD, and *Lactobacillus casei* had better efficacy and medium tolerance in reducing the *Clostridium difficile* infection. Furthermore, the effects on AAD of both a single strain and a combination of strains were equal [10].

*Streptococcus thermophiles*, one of the most considerable traditional fermented starters, is currently considered to have positive health effects [8]. Several clinical trials have reported that products containing *Streptococcus thermophiles* were useful for the treatment and prevention of AAD. A commercial probiotic formula consisting of *Bifidobacterium lactis* and *Streptococcus thermophilus* reduced the frequency of AAD in infants significantly [11]. Probiotic preparation VSL#3 containing *Streptococcus thermophiles* was efficacious in preventing AAD in average-risk patients [12]. Probiotics with *Streptococcus thermophiles* improved the eradication rate of antibiotics to *Helicobacter pylori* and significantly minimized side-effect prevalence such as diarrhea and nausea/vomiting [13]. Additionally, lactic acid from *Streptococcus thermophiles* impacted the progression of *C. difficile* infection, which was present in 20% of AAD patients [14]. Therefore, it is interesting to explore whether *Streptococcus thermophiles* itself has an impact on AAD. 

*Streptococcus thermophiles* DMST-H2 (DMST-H2) was isolated from a Chinese traditional yogurt, and showed potential probiotics. This study aims to evaluate the effects of DMST-H2 in AAD mice models from three aspects: (1) whether it can help to ameliorate the status of diarrhea; (2) whether it can help to repair tissue injury and regulate the inflammatory response; (3) whether it can help to regulate intestinal flora. This study enriches scientific understanding about DMST-H2 and facilitates its further application in antibiotic-associated diarrhea.

## 2. Materials and Methods 

### 2.1. Isolation and Identification of DMST-H2

The Chinese traditional yogurt was purchased from an Inner Mongolia family who makes traditional yogurt with no commercial starter. It was diluted (10^−5^ to 10^−7^) with sterile saline, and 100 μL of the dilution was plated on MRS ager medium (Guangzhou huankai Biotechnology Co., Ltd., Guangzhou, China). After anaerobic was cultured at 37 °C for 48 h, bacterial colonies were purified by re-streaking. The pure strains were cultured overnight in MRS medium (Guangzhou Huankai Biotechnology Co., Ltd., Guangzhou, China) at 37 °C for 16S rDNA identification according to Yi et al. [15]. Each sequence was compared with the National Center for Biotechnology Information (NCBI) using BLAST. A phylogenetic tree was constructed using the neighbor-Joining method [16] in the Molecular Evolutionary Genetics Analysis (MEGA) X 10.1.7 (National Institutes of Health, Bethesda, USA) [17].

### 2.2. Acute Oral Toxicity Test

An acute oral toxicity test of DMST-H2 was carried out by Guangzhou Quality Supervision and Testing Institute (GQT, License number: SYXK 2018-0137). Twenty healthy specific pathogen free Kunming (SPF KM) mice (10 females and 10 males, weight 20.00 ± 2.00 g) were supplied by Ji’nan Peng Yue Laboratory Animal Breeding Co. Ltd. (License number: SCXK 2014-0007). The room temperature was 24 ± 2 °C, and relative humidity was 40–70%. All mice were fasted overnight and then gavaged with DMST-H2 (1 × 10^8^ CFU/mL) with a dose of 10.0 g/kg bw. Experimental observation of toxicity signs and death lasted for 14 days, and the mice were weighed every 7 days [18]. 

### 2.3. Genome Sequencing, Assembly, and Bioinformatic Analyses

Genomic DNA was extracted using a Blood & Cell Culture DNA Midi Kit (Cat. No. 13343, Qiagen, USA) according to manufacturer protocol. DNA concentration and purity were determined via Qubit fluorometer and Nanodrop 2000 spectrophotometer (Thermo Fisher Scientific, Carlsbad, CA, USA). Whole genome sequencing was performed on the MGISEQ-2000 platform and PacBio Sequel system at BGI (Shenzhen, China). The obtained reads were assembled using Falcon v0.3.0 (Pacific Biosciences of California, Inc., Menlo Park, USA), proovread 2.12 (University of Würzburg, Würzburg, Germany), Celera Assembler 8.3 (J. Craig Venter Institute., La Jolla, USA), SMRT Analysis v2.3.0 (Pacific Biosciences of California, Inc., Menlo Park, USA) and GATK v1.6-13 (Broad Institute, Cambridge, USA). Gene prediction was performed using Glimmer v3.02 (University of Maryland Center for Bioinformatics & Computational Biology, USA), RNAmmer 1.2 (Technical University of Denmark, Copenhagen, Denmark), tRNAscan-SE 1.3.1 (The Lowe Lab, Biomolecular Engineering, University of California Santa Cruz, Santa Cruz, USA) and Rfam 9.1 (European Bioinformatics Institute, Cambridge, UK). Functional annotation of genes were searched against the Kyoto Encyclopedia of Genes and Genomes (KEGG) and Clusters of Orthologous Groups (COG). Antibiotic resistance genes were predicted using the Comprehensive Antibiotic Resistance Database (CARD). The Virulence Factors of Pathogenic Bacteria Database (VFDB) was used to identify the virulence factors. The genome sequence is available in the GenBank under the accession number CP063275.

### 2.4. Resistance to Simulated Gastrointestinal Conditions

The resistance of DMST-H2 to simulated gastrointestinal circumstance was tested as previously described [19]. DMST-H2 was pre-cultured overnight in MRS medium (Guangzhou Huankai Biotechnology Co., Ltd., Guangzhou, China) at 37 °C and then inoculated into artificial gastric juice (pH 2.5). One hour later, intestinal solution was added (pH 8.0), and inoculation continued until 3 and 5 h. The viable bacteria was counted by planting method at each time point. The survival rate was calculated as following: Survival rate = final lg (CFU/mL)/initial lg (CFU/mL).

### 2.5. Preparing for DMST-H2 Materials

The freeze-dried DMST-H2 and yogurt fermented with it were used in this study. The freeze-dried DMST-H2 powder was inoculated into 10% sterilized skim milk (Qingdao Nestle Co., Ltd., Qingdao, China) and fermented at 42 °C until solid. Before administration, the viable cell count of DMST-H2 in powder and yogurt were adjusted to 2 × 10^9^ CFU/mL with sterile saline.

### 2.6. Animals and the Experiment Design

**Figure 1:**
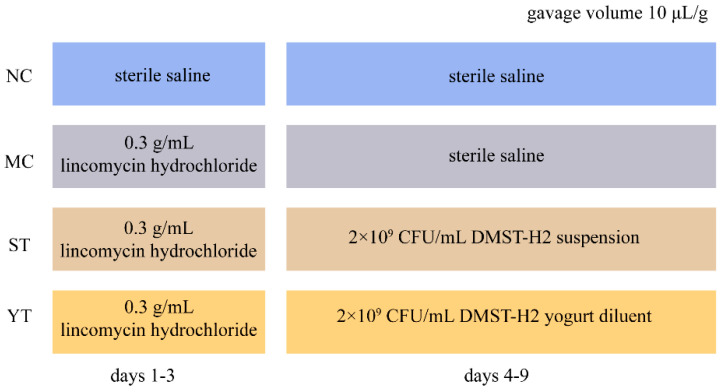
Experimental design schematics. NC, Normal control group (*n* = 8); MC, Model control group (*n* = 8); ST, DMST-H2 treatment group (*n* = 8); YT, yogurt treatment group (*n* = 8).

All animal procedures were performed following the Guidelines for Care and Use of Laboratory Animals of South China Agricultural University (Guangzhou, China, License number: SYXK 2019-0136) and experiments were approved by the Animal Ethics Committee of Southern Medical University (permit number 2019183, 14 November, 2019). SPF Male Balb/c mice (weight 20.00 ± 2.00 g, age 6–8 weeks) were provided by Southern Medical University (Guangzhou, China, License number: SCXK 2016-0041). After one week of adapting (24 ± 2 °C, 45–55% humidity, and normal day/night cycle), the mice were randomly divided into 4 groups with 8 mice per group as shown in Figure 1. Model control (MC) group, DMST-H2 treatment group (ST) and yogurt treatment group (YT) were intragastrically administered lincomycin hydrochloride (0.3 g/mL, Bio Basic Inc., Markham, ON, Canada) for 3 days (10 μL/g, twice a day, days 1–3) [20]. After establishing the AAD model, ST, YT, and MC mice were treated with DMST-H2 suspension, yogurt diluent, and sterile saline respectively for 6 days (10 μL/g, once a day, days 4–9). The normal control (NC) group was treated with sterile saline for 9 days. Twelve hours after the last gavage administration, we collected the blood and obtained serum by centrifugation (1500 rpm, 10 min) [21]. The ileum, cecum and spleen were collected and ileum was stored in 10% formalin (Servicebio Co., Ltd., Wuhan, China). The intestinal contents (from the jejunum to rectum, >0.5 g) were stored in dry ice. Fecal samples from each mouse were collected at the same time every day.

### 2.7. Diarrhea Measurement

Evaluating parameters of diarrhea symptoms were fecal consistency and fecal water content. Fecal consistency was measured on a 3 grade scale: formed, shaped and brown, score = 1; soft, does not pour, yellow, score = 2; liquid, yellow, score = 3 [22,23]. Fecal samples were weighed after collection (fresh fecal weight), and then dried to a constant weight in 95 °C (dried fecal weight). The calculation formula of fecal water content was: Fecal water content = 1 − (dried fecal weight)/(fresh fecal weight).

### 2.8. Feces Bacterial Culture

Fresh feces on days 0 and 3 were diluted into 10^−4^–10^−6^ with sterile saline and then spread onto selective agar plates. TPY medium (Hopebio Co., Ltd., Qingdao, China), LBS medium (Hopebio Co., Ltd., Qingdao, China), bile aesculin azide medium (Hopebio Co., Ltd., Qingdao, China) and EMB (eosin and methylene blue) medium (Barebio Co., Ltd., Hangzhou, China) were used to detect *Bifidobacterium*, *Lactobacillus*, *Enterococcus,* and Enterobacteriaceae, respectively.

### 2.9. Organ Index and Histological Observation

The ileum fixed in formalin was stained by hematoxylin and eosin (HE) [24], and then observed under an Olympus BH22 Microscope (Tokyo, Japan). Cecum index was calculated as follow: Cecum index = cecum weight/body weight.

### 2.10. Enzyme-Linked Immunosorbent Assays of Serum

The levels of tumor necrosis factor-alpha (TNF-α), interleukin-10 (IL-10) and interferon-γ (IFN-γ) were determined using mouse ELISA kits following the manufacturer’s instructions (Neobioscience, Shenzhen, China). Endotoxin (ET), D-lactate (D-LA) and Diamine oxidase (DAO) concentrations were also measured using mouse ELISA kits (Shanghai Enzyme-linked Biotechnology Co. Ltd., Shanghai, China).

### 2.11. Preparation of Total DNA and High Throughput Sequencing Analysis

Microbial DNA was extracted according to HiPure Stool DNA Kits instructions (Magen, Guangzhou, China). The primers used for amplifying the 16S rDNA V3-V4 region were 341F: CCTACGGGNGGCWGCAG, 806R: GGACTACHVGGGTATCTAAT [25]. After being extracted and purified with the AxyPrep DNA Gel Extraction Kit (Axygen Biosciences, Union City, CA, USA), amplicons were quantified using ABI StepOnePlus Real-Time PCR System (Life Technologies, Foster City, USA). Purified amplicons were then pooled in equimolar and paired-end sequenced (2 × 250) on an Illumina platform according to the standard protocols. After being filtered, the effective tags were clustered (similarity above 97%) into operational taxonomic units (OTUs) using UPARSE [26] (version 9.2.64) pipeline.

### 2.12. Bioinformatics and Statistical Analysis

A naive Bayesian model using the (Ribosomal Database Project) RDP classifier 2.2 (Center for Microbial Ecology, Michigan State University, East Lansing, USA) was used to classify the representative sequences [27] based on the Greengene database (version gg_13_5) [28]. Alpha diversity index was calculated in (Quantitative Insights Into Microbial Ecology) QIIME 1.9.1 (QIIME development team, Colorado, USA) [29]. As for beta diversity analysis, sequence alignment was performed using Muscle 3.8.31 (Robert C. Edgar, Mill Valley, USA) [30] and FastTree 2.1 (Lawrence Berkeley National Lab, Berkeley, USA) was used to construct a phylogenetic tree [31]. Furthermore, an unweighted unifrac distance matrix was generated by GuniFrac package 1.0 (University of Colorado, Boulder, USA) [32] in the R project. Principal coordinates analysis (PCoA) of unweighted unifrac was generated in the R project Vegan package (version 2.5.3) [33]. PICRUSt 2.1.4 (Dalhousie University, Halifax, Canada) inferred the KEGG pathway of the OTUs [34]. Microbiological analysis was calculated in the R project Vegan package (version 2.5.3), including a Wilcoxon rank test, Tukey’s HSD test, Kruskal-Wallis H test, and Adonis (also called Permanova) test [33]. Other data were presented as mean ± SD (standard deviation) from at least three independent measurements. The statistical difference was performed using SPSS 16.0 (International Business Machines Co., Amonk, USA) using one-way analysis of variance (ANOVA) followed by least significant difference (LSD) test. Differences were considered significant at *p* < 0.05. Figures were plotted in R project ggplot2 package (version 3.3.2) [35].

## 3. Results

### 3.1. Identification and General Genome Features of DMST-H2

**Figure 2:**
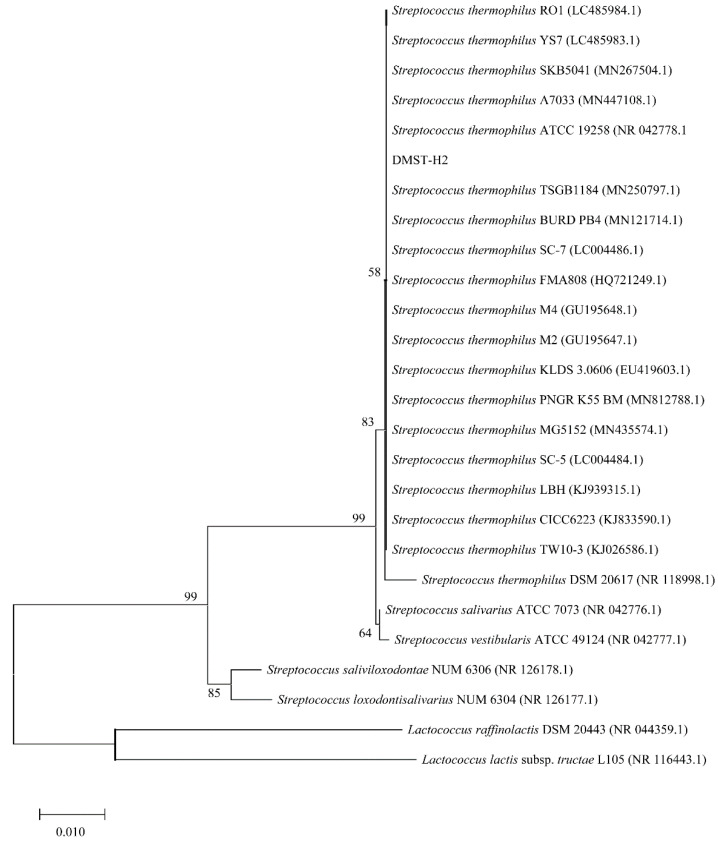
Phylogenetic tree of DMST-H2 16S rRNA gene.

The 16S rDNA of the strain named DMST-H2 showed 99.93% similarity with S*treptococcus thermophilus* ATCC 19258. The phylogenetic tree was showed in Figure 2. It was clear that DMST-H2 had close evolutionary relatedness with S*treptococcus thermophiles* strains, thus DMST-H2 belonged to *Streptococcus thermophiles*.

**Figure 3:**
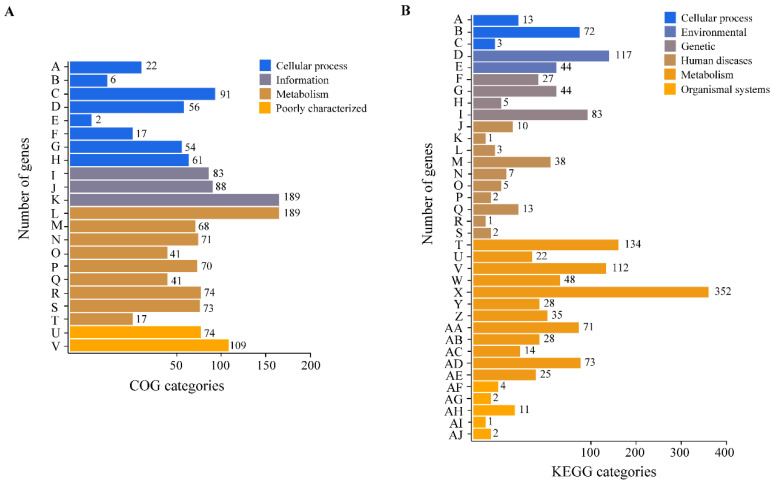
COG and KEGG analysis of DMST-H2. (**A**) Gene number of COG categories. A, Cell cycle control, cell division, chromosome partitioning; B, Cell motility; C, Cell wall/membrane/envelope biogenesis; D, Defense mechanisms; E, Extracellular structures; F, Intracellular trafficking, secretion, and vesicular transport; G, Posttranslational modification, protein turnover, chaperones; H, Signal transduction mechanisms; I, Replication, recombination and repair; J, Transcription; K, Translation, ribosomal structure and biogenesis; L, Amino acid transport and metabolism; M, Carbohydrate transport and metabolism; N, Coenzyme transport and metabolism; O, Energy production and conversion; P, Inorganic ion transport and metabolism; Q, Lipid transport and metabolism; R, Mobilome: prophages, transposons; S, Nucleotide transport and metabolism; T, Secondary metabolites biosynthesis, transport and catabolism; U, Function unknown; V, General function prediction only. (**B**) Gene number of KEGG categories. A, Cell growth and death; B, Cellular community-prokaryotes; C, Transport and catabolism; D, Membrane transport; E, Signal transduction; F, Folding, sorting and degradation; G, Replication and repair; H, Transcription; I, Translation; J, Cancers: Overview; K, Cancers: Specific types; L, Cardiovascular diseases; M, Drug resistance: Antimicrobial; N, Drug resistance: Antineoplastic; O, Endocrine and metabolic diseases; P, Immune diseases; Q, Infectious diseases: Bacterial; R, Infectious diseases: Viral; S, Neurodegenerative diseases; T, Amino acid metabolism; U, Biosynthesis of other secondary metabolites; V, Carbohydrate metabolism; W, Energy metabolism; X, Global and overview maps; Y, Glycan biosynthesis and metabolism; Z, Lipid metabolism; AA, Metabolism of cofactors and vitamins; AB, Metabolism of other amino acids; AC, Metabolism of terpenoids and polyketides; AD, Nucleotide metabolism; AE, Xenobiotics biodegradation and metabolism; AF, Aging; AG, Digestive system; AH, Endocrine system; AI, Immune system; AJ, Nervous system.

DMST-H2 contained a single circular chromosome of 1879014 base pairs (bp), with a G + C% content of 83.24%. A total of 2016 genes were identified with an average length of 775.81 bp. The chromosome harbored 18 rRNAs, 67 tRNAs, and 16 sRNAs, 24 minisatellite DNAs, and 2 microsatellite DNAs. 1497 genes were assigned to COGs, functioning in metabolism (644 genes, 43.02%), information (360 genes, 24.05%), cellular process (309, 20.64%), and poorly characterized (183, 12.22%) (Figure 3A). Furthermore, 1452 genes were classified into KEGG, and most genes were involved in metabolism (942, 64.88%) and genetic information processing (159, 10.95%) (Figure 3B).

### 3.2. Safety Evaluation of DMST-H2

In the acute oral toxicity test, no obvious toxic signs and death were observed. According to the GB 15193.3-2014 [18], the acute oral lethal dose 50% (LD_50_) of DMST-H2 in KM mice was more than 10.0 g/kg bw. Therefore, DMST-H2 can be classified as actual non-toxic grade (Table 1). 

According to CARD annotation, DMST-H2 showed no antibiotic resistance genes. Although a total of 99 (4.91%) genes were identified as putative virulence factor genes, they carried out the functions like amino acid transport and metabolism, nucleotide transport and metabolism, carbohydrate transport and metabolism, transcription, lipid transport and metabolism, translation, ribosomal structure and biogenesis, cell wall/membrane/envelope biogenesis, cell motility, posttranslational modification/protein turnover/chaperones, inorganic ion transport and metabolism, and signal transduction mechanisms according to COG. In fact, they could not be considered really harmful, because they could also represent essential probiotic traits for adhesion and protection [36,37].

### 3.3. Probiotic Potential of DMST-H2

**Figure 4:**
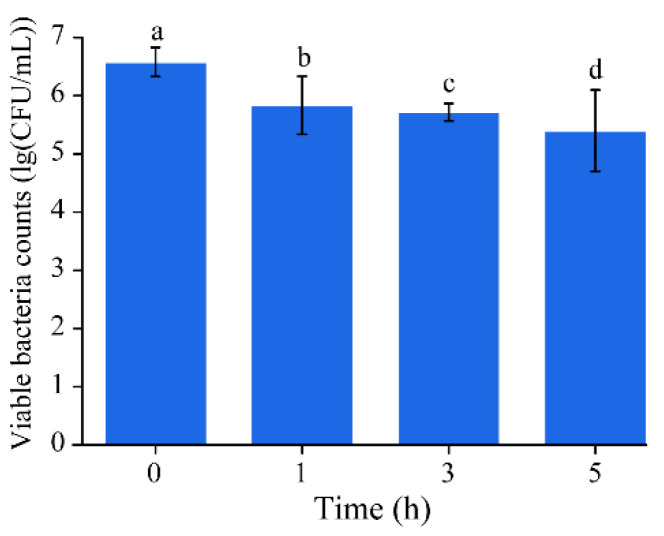
Survival of DMST-H2 in simulated gastrointestinal conditions. Significance was set as *p* < 0.05, and values that do not share a common letter differed significantly (*p* < 0.05).

The resistance of the gastrointestinal tract environment is a key factor for bacterial strains to be considered as probiotics [8]. DMST-H2 presented resistance ability to artificial gastric and intestinal juice since the initial survival was 82.04% (1.18-lg(CFU/mL) decrease, Figure 4). 

Genomic analysis is shown in Table 2. The presence of F1F0 ATPase subunits and tyrosyl-tRNA synthetase genes suggest the ability of acid tolerance [38,39]. Furthermore, DMST-H2 possessed genes encoded with cyclopropane-fatty-acyl-phospholipid synthase [40,41] and choloylglycine hydrolase for bile salt tolerance [42]. 

For protection against reactive oxygen, the strain carried thioredoxin reductase (NADPH), glutathione reductase, peptide methionine sulfoxide reductase msrA/msrB [43], thiol peroxidase, thioredoxin 1 [42], and superoxide dismutase. The genes involved in adhesion in DMST-H2 included sortase A [44], elongation factor Tu, competence protein ComGC [43], and chaperonin GroEL [45]. These findings suggest the strain’s ability to adapt to gastrointestinal tract conditions. Lantibiotic compounds, such as nisin, are natural antibacterial peptides that do not produce bacterial resistance [46]. DMST-H2 equipped 12 genes related to bacteriocin [47], which suggested that this strain may have potential antibacterial ability.

### 3.4. DMST-H2 Reduces AAD-Related Symptoms

**Figure 5:**
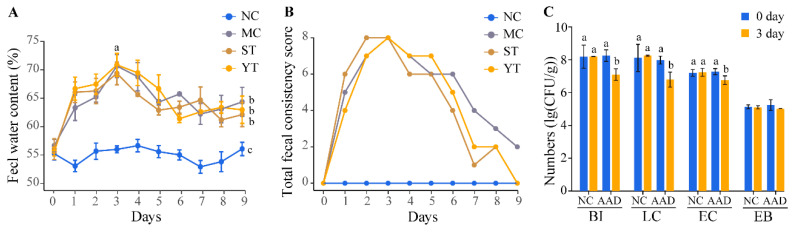
Symptoms of antibiotic-associated diarrhea. (**A**) The transition of fecal water content from each treatment group over the experimental period. (**B**) Total fecal consistency score for each treatment group during the study. (**C**) Fecal culture of normal control mice and antibiotic-associated diarrhea mice pre- and post- lincomycin hydrochloride modeling. BI, *Bifidobacteria*; LC, *Lactobacilli*; EC, *Enterococcus*; EB, Enterobacteriaceae; NC, Normal control group (*n* = 8); MC, Model control group (*n* = 8); ST, DMST-H2 treatment group (*n* = 8); YT, yogurt treatment group (*n* = 8). Significance was set as *p* < 0.05, and values that do not share a common letter differed significantly (*p* < 0.05).

Three days after lincomycin hydrochloride gavage, all mice from the three groups developed soft stools. Fecal water content and fecal consistency scores increased compared with the NC group during this period. On day three, fecal water content in the antibiotic treatment group (MC, ST and YT groups) reached 70.30 ± 2.80%, which was significantly higher than the NC group (55.00 ± 2.23%, *p* < 0.05, Figure 5A). At the same time, total fecal consistency scores reached the maximum value (Figure 5B). Bacterial culture-based assays of *Bifidobacteria*, *Lactobacilli*, *Enterococcus* and Enterobacteriaceae were performed on days zero and three to examine AAD-related intestinal flora imbalance. *Bifidobacteria* and *Lactobacilli* were considered vital components of the gut microbiota and possessed many benefits [48]. While after intragastric administration of lincomycin hydrochloride, viable cell counts of *Bifidobacteria* and *Lactobacilli* declined significantly (*p* < 0.05) with survival rates of only 6.50% and 8.93%, respectively. However, the survival rate of *Enterococcus* and *Enterobacteriaceae* still maintained 57.09% and 67.49%. In addition, the bacteria in the NC group remained relatively stable during the modeling period (Figure 5C). The above results describe that the AAD had been successfully modeled.

After treating with sterile saline (MC group) or DMST-H2 (ST and YT groups), fecal water content obviously decreased (*p* < 0.05), but normal conditions were not restored (*p* > 0.05, Figure 5A). Furthermore, fecal consistency in ST and YT groups returned to the normal level at day 9 (Figure 5B).

### 3.5. DMST-H2 Improved AAD-Related Inflammatory Reaction and Tissue Damage

**Figure 6:**
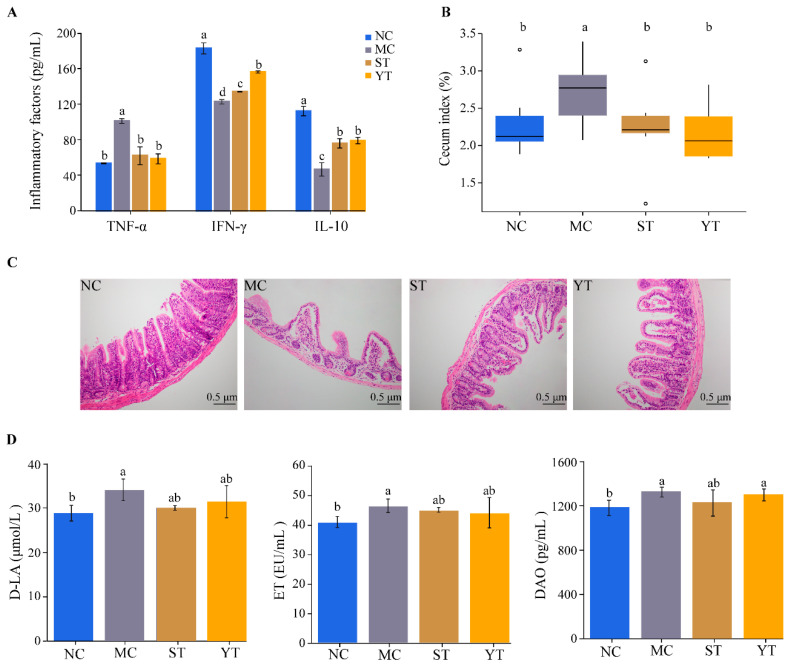
Effect of *Streptococcus thermophiles* DMST-H2 on inflammatory reaction and tissue damage. (**A**) Inflammatory factors (TNF-α, IFN-γ, IL-10) in the serum from each group. (**B**) Cecum index in each group. (**C**) Histological analysis of the ileum (200×). (**D**) Levels of D-LA, ET and DAO. NC, Normal control group (*n* = 8); MC, Model control group (*n* = 8); ST, DMST-H2 treatment group (*n* = 8); YT, yogurt treatment group (*n* = 8). Significance was set as *p* < 0.05, and values that do not share a common letter differed significantly (*p* < 0.05).

AAD was always accompanied by systemic inflammation, which manifested as a significant increase of proinflammatory cytokines and decrease of anti-inflammatory cytokines [49]. In the MC group, the level of TNF-α elevated and IL-10, IFN-γ decreased significantly (*p* < 0.05), which might be related to the systemic inflammation of mice. ST and YT groups increased the content of IL-10 and IFN-γ and decreased the level of TNF-α significantly (*p* < 0.05). Nevertheless, only TNF-α was completely restored to the normal level (*p* > 0.05, Figure 6A).

AAD also induced the pathological injuries of some organs, such as cecal enlargement [50] and intestinal barrier injury. Consistently, the MC group significantly increased the cecum index (2.70 ± 0.46%) compared to the NC group (2.14 ± 0.22%) (*p* < 0.05). ST and YT groups completely cured it with the cecum index dropping to 2.25 ± 0.12% and 2.16 ± 0.37%, respectively (*p* > 0.05, Figure 6B). The ileum pathology slices of each group are shown in Figure 6C. In the NC group, the intestinal villus was structurally intact and closely arranged. But it was short and sparse in the MC group; in addition, the serosa and muscularis became thinner, and inflammatory cell infiltration was observed. ST and YT groups significantly alleviated the pathological features of the ileum, indicated by the smoother and closer villus and fewer inflammatory cells compared to the MC group. At the molecular level, ET, D-LA and DAO were sensitive indexes to detect the damage of intestinal barrier, which increased significantly in MC group (*p* < 0.05) and decreased slightly in ST and YT groups (Figure 6D).

### 3.6. Composition and Difference Analysis of Gut Microbiota

**Figure 7:**
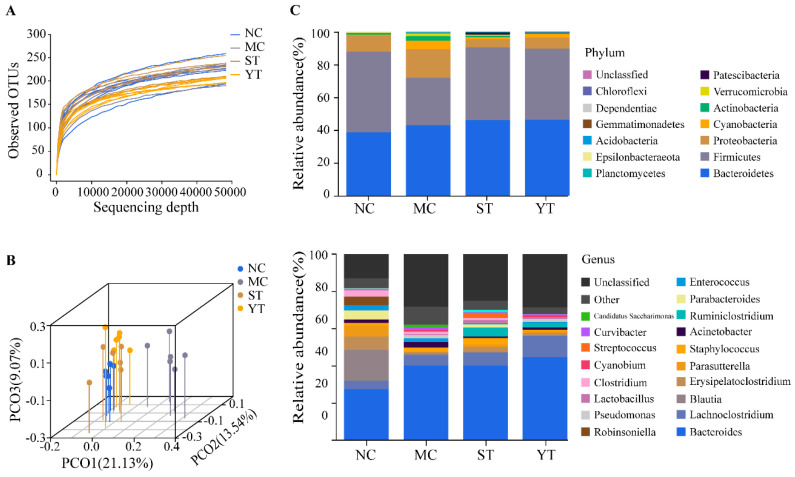
Diversity and composition of Intestinal microbiota in the NC, MC, ST and YT groups. (**A**) Rarefaction curves of observed OTUs for each sample. (**B**) Principal Coordinate Analysis (PCoA) by UniFrac (unweighted) of microbiomes. (**C**) The composition of gut microbiota at the phylum (up) and genus (down) levels. NC, Normal control group (*n* = 6); MC, Model control group (*n* = 6); ST, DMST-H2 treatment group (*n* = 6); YT, yogurt treatment group (*n* = 6).

The end of observed OTUs rarefaction curves were in a flat shape, revealed that the sequencing depth was sufficient for further analysis (Figure 7A). The Simpson and ACE indexes were highest in the NC group but did not differ significantly in each group (*p* > 0.05). Remarkably, the 3D principal component analysis suggested differences among groups (Adonis/Permanova test, R^2^ = 0.2935, *p* = 0.001). The MC group formed a distinctive cluster from the NC, ST, and YT groups. Clusters from ST and YT groups were much closer to the NC group, thereby indicating that the bacterial community structures were more similar between the NC, ST, and YT groups (Figure 7B). 

The composition of intestinal flora is presented in Figure 7C. The predominant phylum were Bacteroidetes, Firmicutes, and Proteobacteria, and the sum of the three contributed 97.86%, 89.22%, 95.28%, and 96.40% to the total bacteria in the NC, MC, ST, and YT groups, respectively. Furthermore, Proteobacteria increased and Firmicutes decreased in the MC group compared to NC, which is consistent with previous findings [20]. The relative proportion of Proteobacteria and Firmicutes recovered in response to DMST-H2 treatment in the ST and YT groups (Figure 7C up panel). An increased prevalence of Proteobacteria [51] and Bacteroidetes/Firmicutes are evidence of gut microbial dysbiosis [52]. In this study, the content of Proteobacteria and Bacteroidetes/Firmicutes (0.78 in NC, 1.52 in MC) increased in the MC group, indicating that lincomycin hydrochloride could cause a dysbiosis of gut microorganism and will persistently continue under self-recovery. However, DMST-H2 could assist with the control of Proteobacteria and Bacteroidetes/Firmicutes (1.04 in ST, 1.06 in YT) and thus regulate the balance of intestinal flora. 

At the genus level, the top 20 abundant genera were shown. Among them, *Bacteroides* (24.98%) and *Blautia* (15.96%) were the dominant genera in the NC group. However, exposure to lincomycin hydrochloride caused higher levels of *Bacteroides* (39.95%, 39.23% and 43.96% respectively) and lower levels of *Blautia* (0.12%, 0.27% and 0.17% respectively) in the MC, ST and YT groups. Compared to the NC group, the genera *Lachnoclostridium*, *Staphylococcus*, *Acinetobacter*, *Pseudomonas* and *Curvibacter* increased in the MC group, which included species involved in pathogenesis. Additionally, genera of beneficial microorganisms *Erysipelatoclostridium*, *Parasutterella* and *Parabacteroides* were limited in the MC group. As for the ST and YT groups, *Lachnoclostridium*, *Ruminiclostridium*_5, and *Streptococcus* (annotated as *Streptococcus salivarius* subsp *thermophilus*) increased (Figure 7C down panel).

**Figure 8:**
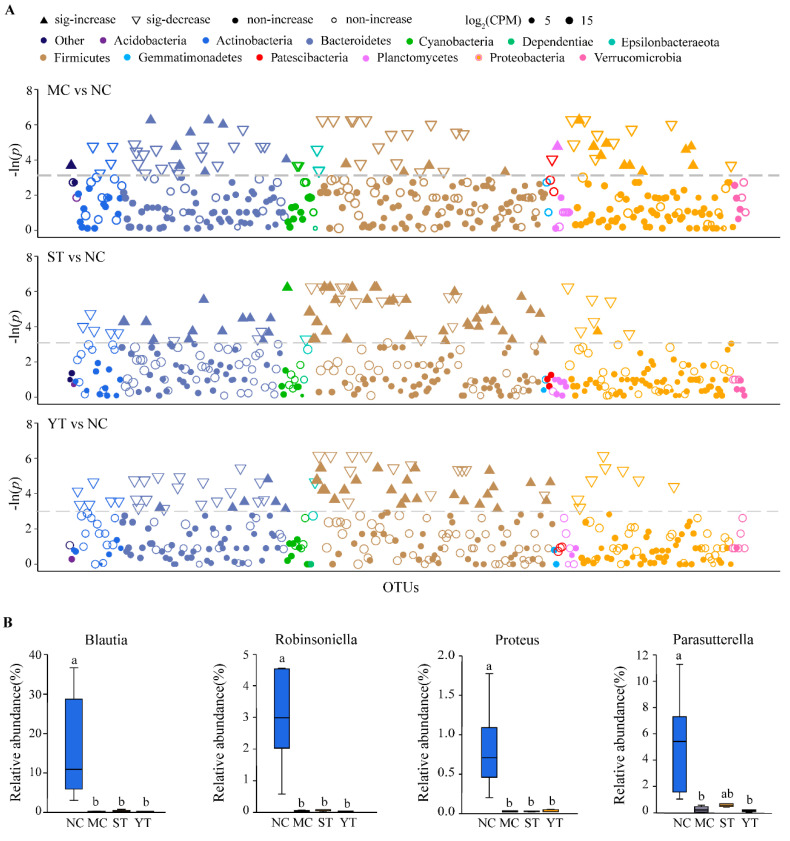
Different microbiota among the groups. (**A**) Manhattan plots showing enriched and depleted OTUs in MC vs NC, ST vs NC and YT vs NC at the phylum level. The dashed line corresponds to the false discovery rate-corrected *p* value (FDR) threshold of significance (Wilcoxon test, α = 0.05). The size of the point represents the relative abundance of the OTUs. The point identifies the type of changes, the shape of the solid triangle represents increased enrichment, hollow triangles represent the cut depleted, solid dots indicate increased with no significant difference and hollow dots indicate decreased with no significant difference. (**B**) Main different compositions at the genus level among NC, MC, ST and YT. Kruskal-Wallis test, Tukey HSD, FDR <0.05. NC, Normal control group (*n* = 6); MC, Model control group (*n* = 6); ST, DMST-H2 treatment group (*n* = 6); YT, yogurt treatment group (*n* = 6). Significance was set as *p* < 0.05, and values that do not share a common letter differed significantly (*p* < 0.05).

To further investigate the differences in bacteria species of these four groups, the relative abundances of all OTUs among the groups were compared in Figure 8. We found 19.52–21.74% percent of OTUs still significantly differed between the NC and AAD groups (MC, ST and YT groups, *p* < 0.05). Though the number of different OTUs in the DMST-H2-treated groups (ST and YT) and MC group were similar, the species were distinguished. The significant highly abundant OTUs in the MC group were mainly within the phylum of Proteobacteria (genus *Pseudomonas*, *Anaeromyxobacter*, *Rodentibacter*, *Sphingomonadaceae*, *Mitochondria*, *Stenotrophomonas*, *Ralstonia*), and Bacteroidetes (genus *Muribaculaceae* and *Bacteroides*). Moreover, the highly decreased OTUs were within the phylum of Firmicutes (genus *Blautia*, *Robinsoniella*, *Kurthia*, *Aerococcus*, *Erysipelatoclostridium*, *Lactobacillus*), and Actinobacteria (genus *Glutamicibacter*, *Bifidobacterium*, *Enterorhabdus*) (Figure 8A up penal, *p* < 0.05). In addition, OTUs markedly enriched in the ST group belonged to the phylum of Firmicutes (genus *Lachnoclostridium*, *Ruminiclostridium*_5, *Streptococcus*, *Erysipelatoclostridium*, Candidatus *Stoquefichus*), and Bacteroidetes (genus *Bacteroides*, *Parabacteroides*), and decreased OTUs were mainly within the phylum of Actinobacteria (genus *Corynebacterium*_1, *Glutamicibacter*) and Proteobacteria (genus *Parasutterella*, *Proteus*) (Figure 8A middle penal, *p* < 0.05). In YT groups, the significant highly abundant OTUs were mainly within the phylum of Firmicutes (genus *Lachnoclostridium*, *Erysipelatoclostridium*), decreased OTUs were mainly within the phylum of Actinobacteria (*Streptomycetaceae*, *Glutamicibacter*, *Corynebacterium*_1), Bacteroidetes (genus *Bacteroides*, *Parabacteroides*), and Proteobacteria (genus *Parasutterella*, *Proteus*) (Figure 8A down penal, *p* < 0.05). After the statistics, a total of five genera were found to be significantly different between the ST and FY groups. Specifically, the ST group showed drastically higher levels of *Parasutterella*, *Parabacteroides*, *Lachnospiraceae*_NK4A136_group, and *Sphingomonas*, while a higher content of *Coprobacillus* was measured in the YT group. Furthermore, four-component analyses were also performed by the Kruskal-Wallis test, reveling that marked reductions in relative abundances of genera *Blautia*, *Robinsoniella*, *Proteus* and *Parasutterella* were noted in MC, ST and YT group compared to NC (Figure 8B, *p* < 0.05). 

The above results showed that lincomycin hydrochloride treatment changed the composition of gut microbiota in various taxons. Natural recovery (MC group) resulting in the limited growth of Firmicutes, and the overgrowth of Proteobacteria which was generally regarded as a characteristic of dysbiosis. However, both ST and YT groups exhibited the opposite and performed a more similar population structure with the NC group. Therefore, DMST-H2 contributes to the recovery of ADD-induced intestinal dysbacteriosis.

### 3.7. Functions Predicted

**Figure 9:**
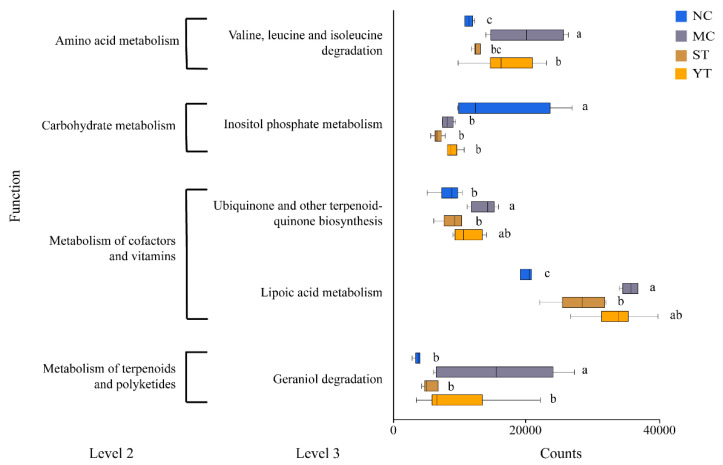
The predicted function of the fecal 16S metabolic pathway (significantly different in level 3 of the metabolic pathway). NC, Normal control group (*n* = 6); MC, Model control group (*n* = 6); ST, DMST-H2 treatment group (*n* = 6); YT, yogurt treatment group (*n* = 6). Significance was set as *p* < 0.05, and values that do not share a common letter differed significantly (*p* < 0.05).

PICRUSt 2 predicted the metabolic processes of gut microbiota. The low value (0.08–0.22) of the nearest sequenced taxon index value (NSTI) indicates the accurate prediction [53]. Figure 9 shows that antibiotic treatment significantly affected the metabolism of amino acids, carbohydrates, cofactor and vitamins, terpenoid, and polyketides. In the MC group, significant increases were found in valine, leucine, and isoleucine degradation, ubiquinone and other terpenoid-quinone biosynthesis, lipoic acid metabolism, and geraniol degradation. Also, inositol phosphate metabolism level decreased. DMST-H2 only recovered part of them.

## 4. Discussion

In this study, we isolated *Streptococcus thermophiles* DMST-H2 from Chinese traditional yogurt. Genomic analysis and in vitro experimentation suggested that DMST-H2 had the potential ability to survive and adhere in the gastrointestinal tract, indicating the possibility of use as a probiotic. We successfully established the AAD mice model based on the results of increased fecal water content and fecal consistency score. Also, an even larger decrease in beneficial bacteria, such as *Bifidobacteria* and *Lactobacilli*, than the decrease in pathogenic bacteria like *Enterococcus* and Enterobacteriaceae was other evidence. Both natural recovery (MC group) and DMST-H2 treatment (ST and YT) groups decreased fecal water content significantly. However, DMST-H2 supplementation performed better at decreasing systemic inflammation, recovering intestinal injury and regulating the changes of intestinal flora.

A growing body of evidence suggests that probiotics are prospective to prevent and treat AAD [3]. Previous studies have found that the high fecal water content may self-reduce as low as the treatment group. Conversely, the treatment group had better outcomes when evaluating intrinsic indicators such as gut barrier integrity and intestinal microbiological changes [20,21,22,23]. Ling et al. indicated that *Clostridium butyricum* and *Bifidobacterium infantis* could relieve systemic inflammation in the AAD mice by returning IL-10, IFN-γ, and TNF-α to normal levels [49]. Besides, a number of in vitro studies have investigated that *S. thermophilus* strains were able to modulate the immune response of various human cell lines [54,55,56]. Our results also showed that DMST-H2 alleviated the inflammatory response by decreasing TNF-α and increasing IFN-γ and IL-10. DMST-H2 harbors gene encoding superoxide dismutase (SOD) antioxidant enzymes (DMST-H2GL000768) which may explain its anti-inflammatory activity according to del Carmen et al. [57]. Work by Del Piano et al. suggested that *S. thermophilus* improved intestinal barrier function [58]. In addition, another study showed that *S. thermophilus* both prevented occludin degradation, rupture of tight cell junctions induced by *E. coli* in vitro, and decreased epithelial cell death [59]. Additionally, it also prevents bacterial translocation in colitic animals [60]. Consistent with findings of these studies, supplementation with DMST-H2 significantly recovered intestinal injury. 

In the case of dysbacteriosis, probiotics supplements could have a significant impact on reshaping the microbiota. Plenty of research on the AAD mice/rat model presented the same change of gut flora after antibiotic treatment: lower abundances of Firmicutes and over-representation of Proteobacteria [61,62,63]. Our results also showed the same trend and DMST-H2 increased Firmicutes and decreased Proteobacteria successfully. DMST-H2 restored the gut flora closer to the normal control group. However, it was difficult to recover the decreased content of genus *Blautia* and *Parasutterella*. *Blautia* could produce butyric acid, and butyrate will benefit the intestinal mucosa repair, increase the expression of ZO-1, and decrease the gut endotoxin levels in serum [64]. *Parasutterella* is a core component of the human gut microbiota [65] and plays a potential role in bile acid maintenance and cholesterol metabolism [66]. Since the gut microbiome is considered an organ contributing to the regulation of host metabolism [67], the change of gut microflora leads to a change of metabolic pathway. For example, Li et al. [20] and our results show that there was an increase in the metabolism of amino acids in the presence of AAD.

## 5. Conclusions

Taken collectively, this study acquired a non-oral toxicity probiotic strain *Streptococcus thermophiles* DMST-H2, which is equipped with putative genes for adapting to gut transit stresses and showed tolerance to simulated gastrointestinal fluid in vivo. The animal experiment indicated that DMST-H2 had a potent effect on promoting recovery in AAD mice compared to natural recovery, and demonstrated it from three aspects: (1) DMST-H2 relieved diarrhea symptoms effectively, manifested in the reduction of fecal water content and fecal consistency score; (2) DMST-H2 positively recovered the inflammation and intestinal injury induced by AAD. TNF-α decreased while IL-10 and IFN-γ increased. Also, DMST-H2 lowered the cecal index, improved the intestinal barrier injury, and reduced ET, D-LA and DAO content in serum; (3) DMST-H2 better restored the microbial environment in the guts of mice with AAD, and the bacterial structures were much closer to the natural control group. Therefore, DMST-H2 deserves further research on AAD treatment. The joint analysis of microbiome and metabolomics will be carried out in further research for the purpose of analyzing the functionary mechanism of DMST-H2.

## Figures and Tables

**Table 1:** The result of acute oral toxicity test.

Sex	Weight (g)			Death Rate (%)
	Day 0	Day 7	Day 14	
Female	19.08 ± 0.76	27.00 ± 1.53	32.08 ± 1.54	0
Male	20.22 ± 1.60	31.61 ± 2.50	38.50 ± 2.80	0

**Table 2:** Putative genes for probiotic properties in DMST-H2.

Putative Function	Protein Name	Genome Location
Acid tolerance	F0F1-ATPase	DMST-H2GL000469, DMST-H2GL000470, DMST-H2GL000471, DMST-H2GL000472, DMST-H2GL000473, DMST-H2GL000474, DMST-H2GL000475, DMST-H2GL000476
	Tyrosyl-tRNA synthetase	DMST-H2GL001052, DMST-H2GL001867
Bile salt tolerance	Cyclopropane-fatty-acyl-phospholipid synthase	DMST-H2GL000126
	Choloylglycine hydrolase	DMST-H2GL001522
Oxidative stress	Peptide methionine sulfoxide reductase msrA/msrB	DMST-H2GL001340
	Glutathione reductase	DMST-H2GL000398
	Thioredoxin reductase (NADPH)	DMST-H2GL001650
	Superoxide dismutase, Fe-Mn family	DMST-H2GL000768
	Thiol peroxidase, atypical 2-Cys peroxiredoxin	DMST-H2GL001000
	Thioredoxin 1	DMST-H2GL001797
Adhesion	Sortase A	DMST-H2GL001272
	Elongation factor Tu	DMST-H2GL000478
	Chaperonin GroEL	DMST-H2GL000203
	Competence protein ComGC	DMST-H2GL001860
Bacteriocin	Lantibiotic biosynthesis protein	DMST-H2GL000092, DMST-H2GL000093, DMST-H2GL000094
	Lantibiotic biosynthesis response regulator NisR/SpaR	DMST-H2GL000928
	Lantibiotic biosynthesis sensor histidine kinase NisK/SpaK	DMST-H2GL000929
	Bacteriocin exporter	DMST-H2GL000265, DMST-H2GL000267, DMST-H2GL000268, DMST-H2GL000694, DMST-H2GL000695, DMST-H2GL001682, DMST-H2GL001683
